# Exploratory identification of candidate biomarkers and molecular contributors to paclitaxel-induced peripheral neuropathy in patients with breast cancer through proteomic analysis

**DOI:** 10.3389/fpain.2026.1813700

**Published:** 2026-06-16

**Authors:** Nobuyoshi Kittaka, Hideaki Tahara, Takashi Akazawa, Yu Mizote, Takashi Kitajima, Keiichi Minami, Yurika Sugano, Takahiro Nakayama

**Affiliations:** 1 Department of Breast Surgery, Osaka Rosai Hospital, Sakai-shi, Osaka, Japan; 2 Department of Breast and Endocrine Surgery, Osaka International Cancer Institute, Chuo-ku, Osaka, Japan; 3 Department of Cancer Drug Discovery and Development, Research Center, Osaka International Cancer Institute, Chuo-ku, Osaka, Japan; 4 Center for Clinical Research, Osaka International Cancer Institute, Chuo-ku, Osaka, Japan; 5 Translational Research, Ono Pharmaceutical Co., Ltd., Mishima-gun, Osaka, Japan; 6 Drug Discovery Technology, Ono Pharmaceutical Co., Ltd., Mishima-gun, Osaka, Japan

**Keywords:** biomarkers, breast cancer, chemotherapy-induced peripheral neuropathy, proteomics, therapeutic targets

## Abstract

**Background:**

Chemotherapy-induced peripheral neuropathy (CIPN) is a significant adverse effect of paclitaxel in patients with breast cancer. However, reliable biomarkers for early detection and monitoring of CIPN are lacking.

**Methods:**

We investigated neurofilament light chain (NfL) and galectin-3 as candidate biomarkers for CIPN, and performed proteomic analysis to identify novel molecular contributors in two cohorts of patients with breast cancer undergoing paclitaxel therapy. Blood samples were collected longitudinally to measure NfL and galectin-3 levels. Differentially expressed proteins associated with CIPN onset were identified by proteomics.

**Results:**

NfL levels significantly increased in patients with CIPN, from as early as 3 weeks after treatment initiation. NfL levels were markedly higher in patients with vs. without CIPN. There were no changes or between-group differences in galectin-3 levels. Proteomic analysis identified upregulation of alcohol dehydrogenase 4 (ADH4) and downregulation of vesicular overexpressed in cancer prosurvival protein 1 (VOPP1) in CIPN, implicating oxidative stress and disrupted mitochondrial dynamics in the pathogenesis of CIPN.

**Conclusion:**

NfL represents a candidate biomarker for early detection of CIPN, and we identified proteins potentially associated with the development of CIPN. Our findings provide further insights into the pathogenesis of CIPN. Integrating biomarkers and proteomics paves the way for precision medicine to manage chemotherapy-induced toxicities.

## 1 Introduction

Breast cancer is one of the most prevalent malignancies among females worldwide, accounting for ∼2.3 million new cases annually and ∼685,000 deaths in 2020 alone (1). Among the diverse therapeutic options, taxane-based chemotherapies, including paclitaxel, are considered cornerstone treatments due to their established efficacy in reducing recurrence and improving overall survival rates (2). The mechanism of action of paclitaxel involves targeting microtubule stabilization and disrupting cellular mitosis, making it highly effective against rapidly proliferating tumor cells (3).

Despite its effectiveness, paclitaxel is associated with severe adverse effects, particularly chemotherapy-induced peripheral neuropathy (CIPN). CIPN manifests as sensory disturbances, pain, and motor impairment. It significantly impairs the patient's quality of life and often necessitates dose reductions or treatment discontinuation (4). This toxicity is a major obstacle to optimizing the treatment outcomes of patients with breast cancer.

Previous studies have extensively reported the prevalence of CIPN, with rates ranging from 12.1% to 96.2%, depending on the mean/cumulative dose, cancer type, and treatment regimen (5). Genetic predisposition, including polymorphisms in drug-metabolizing enzymes, has been implicated in the differential susceptibility to CIPN in patients treated with paclitaxel (6). Furthermore, some clinical strategies, such as dose modification and supportive care, showed limited success in mitigating symptoms (7).

Animal models and early-phase clinical trials have provided some insights into the mechanisms underlying CIPN, which include mitochondrial dysfunction, neuroinflammation, and axonal degeneration (8). However, translating these findings into effective preventive or therapeutic interventions remains a significant challenge.

Recent research has revealed the involvement of neurofilament light chain (NfL) and galectin-3 in CIPN. Elevated levels of NfL, a structural component of neuronal axons, were detected in the cerebrospinal fluid and blood of patients experiencing CIPN (9). NfL is considered a biomarker of axonal damage and neuronal degeneration, offering an exploratory tool for early detection and severity assessment of CIPN (9). Galectin-3, a carbohydrate-binding protein, plays a role in modulating neuroinflammation and repair processes. Galectin-3 has been implicated in CIPN owing to its effects on glial cell activation and inflammatory pathways, contributing to nerve damage and chronic pain (10). These findings underscore the multifactorial nature of CIPN, with various proteins and pathways contributing to its pathogenesis. Although NfL and galectin-3 both show promise as biomarkers or therapeutic targets, their exact roles in modulating CIPN and their potential clinical applications require further exploration.

In this exploratory study, we investigated NfL and galectin-3 as candidate biomarkers for the onset of CIPN in patients with breast cancer treated with paclitaxel. We also integrated advanced proteomics and bioinformatics approaches to identify previously unrecognized molecules involved in CIPN.

## 2 Methods

### 2.1 Ethics

This study was approved by the Ethics Committee of Osaka International Cancer Institute (approval numbers: 19196 and 22101). The study complied with the Declaration of Helsinki and the Ethical Guidelines for Medical and Biological Research Involving Human Subjects in Japan.

### 2.2 Patients

Patients aged ≥20 years with breast cancer were invited to participate in the study and allocated into one of two cohorts. All patients provided written informed consent to participate in the study.

#### 2.2.1 Cohort 1

Patients were eligible for Cohort 1 if they were scheduled to start paclitaxel or paclitaxel (albumin-suspended)-containing chemotherapy for breast cancer, had a life expectancy of ≥6 months, and had an Eastern Cooperative Oncology Group performance status of 0 or 1. Patients in this cohort could receive concomitant treatment with other anticancer drugs [e.g., trastuzumab, pertuzumab, and bevacizumab (combinational therapies); or cyclophosphamide, fluorouracil, doxorubicin, epirubicin (sequential therapies)] and granulocyte colony-stimulating factor (e.g., pegfilgrastim), but not chemotherapy with a taxane or platinum drug. Patients who had received taxane or platinum chemotherapy within 12 months before the study, patients who were scheduled to undergo surgery within 4 weeks of starting paclitaxel, patients with brain metastases who required treatment or had symptoms, and patients with symptoms of peripheral neuropathy were excluded from the study. Additionally, patients could be excluded if the investigator considered that participation in the study may pose a risk of worsening the patient's medical condition.

#### 2.2.2 Cohort 2

Cohort 2 included patients who were currently receiving paclitaxel and those who had completed treatment based on the clinical assumption that biomarkers associated with the onset of CIPN may remain relevant both during and after treatment, since CIPN can develop during or after the completion of treatment in clinical practice. Therefore, patients were eligible for Cohort 2 if they had received taxane-based chemotherapy for breast cancer, or were currently receiving taxane chemotherapy, and had peripheral sensory neuropathy (PSN) of grade ≥2 according to the Common Terminology Criteria for Adverse Events (CTCAE) v5.0 Japan Clinical Oncology Group (JCOG) at the time of consent. Patients with peripheral neuropathy not caused/related to chemotherapy (e.g., diabetes, uremia, collagen disease, vitamin B1 deficiency, neuromuscular diseases) or hand-foot syndrome, and patients with CIPN caused by previous treatment with a non-taxane anticancer drug were excluded from the study. Additionally, patients could be excluded if the investigator considered that participation in the study may pose a risk of worsening the patient's medical condition. In Cohort 2, the median interval between treatment completion and study enrollment was 379.5 days (range, 23–1,649 days), indicating that most patients were enrolled several months to years after the completion of therapy.

### 2.3 Study design

**Figure 1:**
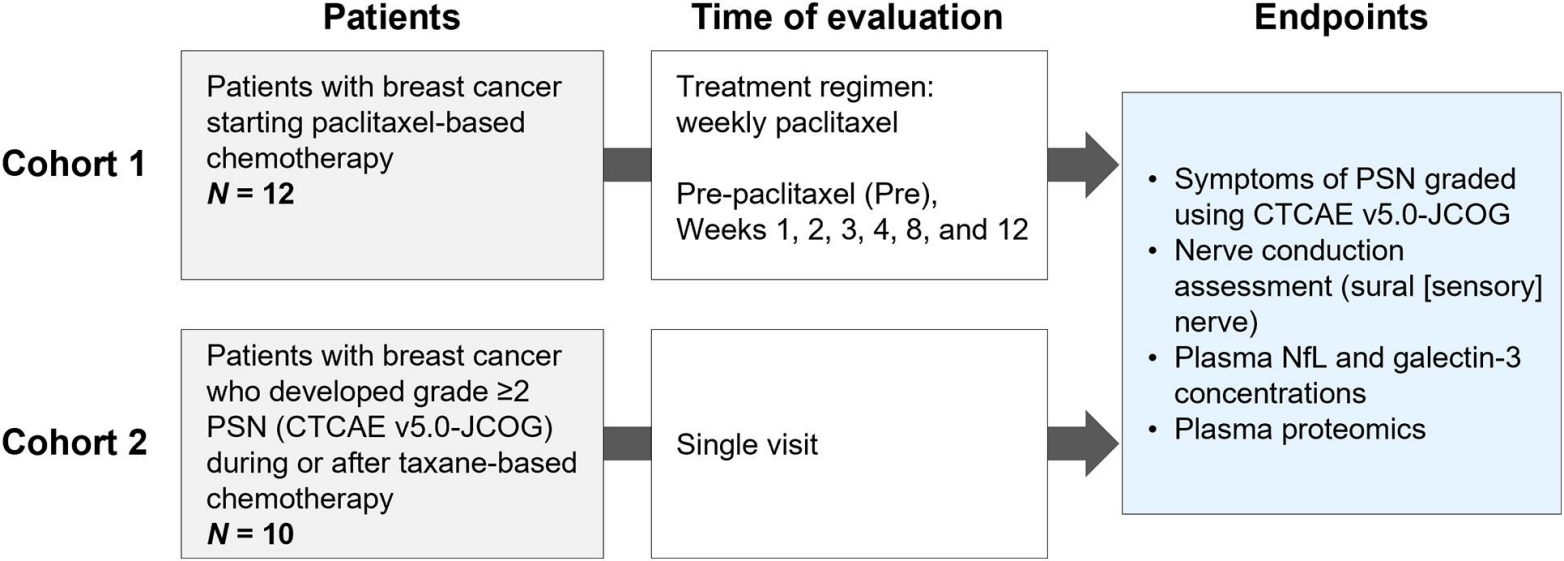
Study design and patient eligibility criteria for cohorts 1 and 2. CTCAE, Common Terminology Criteria for Adverse Events; JCOG, Japan Clinical Oncology Group; NfL, neurofilament light chain; PSN, peripheral sensory neuropathy.

Figure 1 summarizes the study design and endpoints. Patients in Cohort 1 visited the clinic at the following times: before starting weekly paclitaxel, and at 1, 2, 3, 4, 8, and 12 weeks during treatment. Patients in Cohort 2 underwent the study assessments at a single visit. At each visit in Cohort 1 and at the single visit in Cohort 2, a blood sample (5 mL) was collected, and the symptoms of PSN were assessed and graded according to CTCAE v5.0 JCOG criteria. Nerve conduction was assessed at each visit in Cohort 1 and at the single visit in Cohort 2.

### 2.4 Nerve conduction

A DPN-Check® HDN-1000 (Neurometrix Inc.) was used to measure the sensory nerve conduction velocity (SNCV) and sensory nerve action potential (SNAP) of the sural nerve.

### 2.5 Collection and preparation of plasma samples

Peripheral venous blood (approximately 5 mL) was collected. In Cohort 1, blood samples to be used as controls (pre-treatment) were collected between the time of obtaining consent and before the start of paclitaxel administration. Subsequent samples were collected before the start of paclitaxel administration in each cycle. In Cohort 2, blood samples were collected at the single visit.

### 2.6 Measurement of NfL and galectin-3 levels

The plasma NfL levels were quantified using the Simoa NF-light V2 Advantage Kit (Quanterix) with a high-sensitivity automated enzyme-linked immunosorbent assay (ELISA) (Simoa HD-X). The plasma galectin-3 levels were quantified using the Quantikine ELISA Human Galectin-3 Immunoassay (R&D Systems, Inc.) with a fluorescence plate reader (SPECTRAmax iD3; RRID: SCR_023920). The lower limits of quantification were 3 pg/mL for NfL and 0.313 ng/mL for galectin-3. Assays were performed at a contract laboratory (Sekisui Medical Co., Ltd.).

### 2.7 Proteome analysis

Approximately 7,500 proteins were comprehensively quantified using 55 μL of plasma with the SOMAscan platform (SomaLogic Operating Co., Inc.), which is based on aptamers that specifically bind to proteins (11, 12). Using data that had been confirmed to have no quality control problem at the time of measurement, 75th percentile normalization was performed, followed by data distribution and principal component analysis. The subsequent analyses were performed after confirming that there were no clear outliers. To identify proteins that specifically changed in patients with CIPN, Cohort 1 was divided into two groups according to whether they did or did not develop CIPN during treatment with paclitaxel, and the changes in protein expression were analyzed and compared between these groups. In each group, proteins that changed at 4, 8, and 12 weeks relative to before paclitaxel administration (pre-treatment) were extracted as those with a fold-change of ≥1.2 (upregulated) or ≤0.83 (downregulated) in all cases in the group and a *P*-value of <0.05 (paired *t*-test), which were determined using Genedata Profiler (Genedata AG). *Q*-values were calculated simultaneously using the Benjamini–Hochberg and Storey methods. The extracted proteins were compared between the CIPN and non-CIPN groups. Because this analysis was conducted in an exploratory manner and candidate selection was based on nominal *P*-values and fold-changes without controlling for the false discovery rate, false positives may be included in the results. Proteomics data were deposited in the NCBI's Gene Expression Omnibus (GEO) (accession number GSE315782).

### 2.8 Statistical analysis

The data are expressed as the mean ± standard deviation or standard error. Student's *t*-test was used to compare the means between the two groups at each timepoint and before treatment in Cohort 1. Multiplicity across timepoints was adjusted using the Holm method. Changes in NfL and galectin-3 levels were compared between the two groups using the mixed model for repeated measures (MMRM), which included group, time, and an interaction of group × time as fixed factors, and subject as a random effect. When the group effect or the group × time interaction was significant in the MMRM, between-group comparisons at each timepoint were performed using the MMRM, and we used the Holm method to adjust *P*-values for multiple comparisons across timepoints. *P*-values of <0.05 were considered statistically significant (two-sided). Microsoft Excel 2019 (Microsoft Corporation; RRID: SCR_016137) was used to calculate the means, standard deviations, and standard errors, and to create tables. Regression analysis was performed using GraphPad Prism Ver. 8.3.0 (GraphPad Software Inc.; RRID: SCR_002798) with the Japanese add-on (MDF Co., Ltd.). SAS 9.4 (SAS Institute Japan Co., Ltd.; RRID: SCR_008567) and its integrated system EXSUS Ver. 10.1.3 (EPS Co., Ltd.) were used for significance testing.

## 3 Results

### 3.1 Patients

Cohort 1 comprised 12 patients who started treatment with paclitaxel, and Cohort 2 comprised 10 patients who experienced grade ≥2 PSN during or after taxane-based chemotherapy for breast cancer (Figure 1). The baseline characteristics of both cohorts are summarized in Supplementary Table S1. The median (range) ages of Cohorts 1 and 2 were 44 (30‒53) years and 62 (50‒75) years, respectively. All patients had a performance status of 0. None of the patients had a diagnosis of diabetes mellitus. Disease stage ranged from I to III; none of the patients had stage IV breast cancer. There were four patients in each cohort with estrogen receptor-positive breast cancer, two patients in each cohort with progesterone receptor-positive breast cancer, and seven patients in Cohort 2 with HER2-positive breast cancer. None of the patients had previously received taxane- or platinum-based systemic therapy.

### 3.2 Incidence of CIPN in Cohort 1

Out of 12 patients who started treatment with paclitaxel in Cohort 1, six developed CIPN (#01, #03, #04, #05, #09, and #11; Table 1). Of these, two patients progressed to grade 2 CIPN, as defined by CTCAE. Regarding the temporal onset of CIPN following the initiation of paclitaxel treatment, one patient exhibited symptoms at 3 weeks, two patients at 4 weeks, five patients at 8 weeks, and three patients at 12 weeks. The other six patients (#02, #06, #07, #08, #10, and #12) did not develop CIPN during the study period. In terms of nerve conduction (Supplementary Figures S1A,B), the SNCV remained unchanged during the study period in Cohort 1 and was similar to that in Cohort 2. By comparison, there was large variability in the SNAP in Cohort 1, which made it difficult to evaluate its time-course. However, the SNAP was numerically lower in Cohort 2 than at any time in Cohort 1. SNCV and SNAP were not correlated with the grades of PSN (*r* = −0.12 and −0.32, respectively) or peripheral motor neuropathy (*r* = −0.13 and −0.34, respectively).

**Table 1:** Timing of onset and CTCAE grade of peripheral sensory neuropathy and peripheral motor neuropathy in individual patients in Cohort 1.

Patient #	Type of neuropathy	Timepoint						
		Pre	1 W	2 W	3 W	4 W	8 W	12 W
01	Sensory	0	0	0	0	0	**1**	**2**
	Motor	0	0	0	0	0	0	**1**
02	Sensory	0	0	0	0	0	0	0
	Motor	0	0	0	0	0	0	0
03	Sensory	0	0	0	0	0	**1**	0
	Motor	0	0	0	0	0	0	0
04	Sensory	0	0	0	0	0	**1**	–
	Motor	0	0	0	0	0	0	–
05	Sensory	0	0	0	0	**1**	**1**	0
	Motor	0	0	0	0	0	0	0
06	Sensory	0	0	0	0	0	–	–
	Motor	0	0	0	0	0	–	–
07	Sensory	0	0	0	0	0	0	0
	Motor	0	0	0	0	0	0	0
08	Sensory	0	0	0	0	0	–	–
	Motor	0	0	0	0	0	–	–
09	Sensory	0	0	0	0	0	0	**1**
	Motor	0	0	0	0	0	0	0
10	Sensory	0	0	0	0	0	0	0
	Motor	0	0	0	0	0	0	0
11	Sensory	0	0	0	**1**	**1**	**1**	**2**
	Motor	0	0	0	0	0	**1**	0
12	Sensory	0	0	0	0	0	0	0
	Motor	0	0	0	0	0	0	0

Peripheral neuropathy was evaluated and graded according to CTCAE for peripheral sensory neuropathy and peripheral motor neuropathy. Values are reported as the CTCAE grade, with bold font for grade ≥1.

CTCAE, Common Terminology Criteria for Adverse Events; Pre, pre-treatment; W, week.

### 3.3 Evaluation of NfL as a candidate biomarker in Cohorts 1 and 2

**Figure 2:**
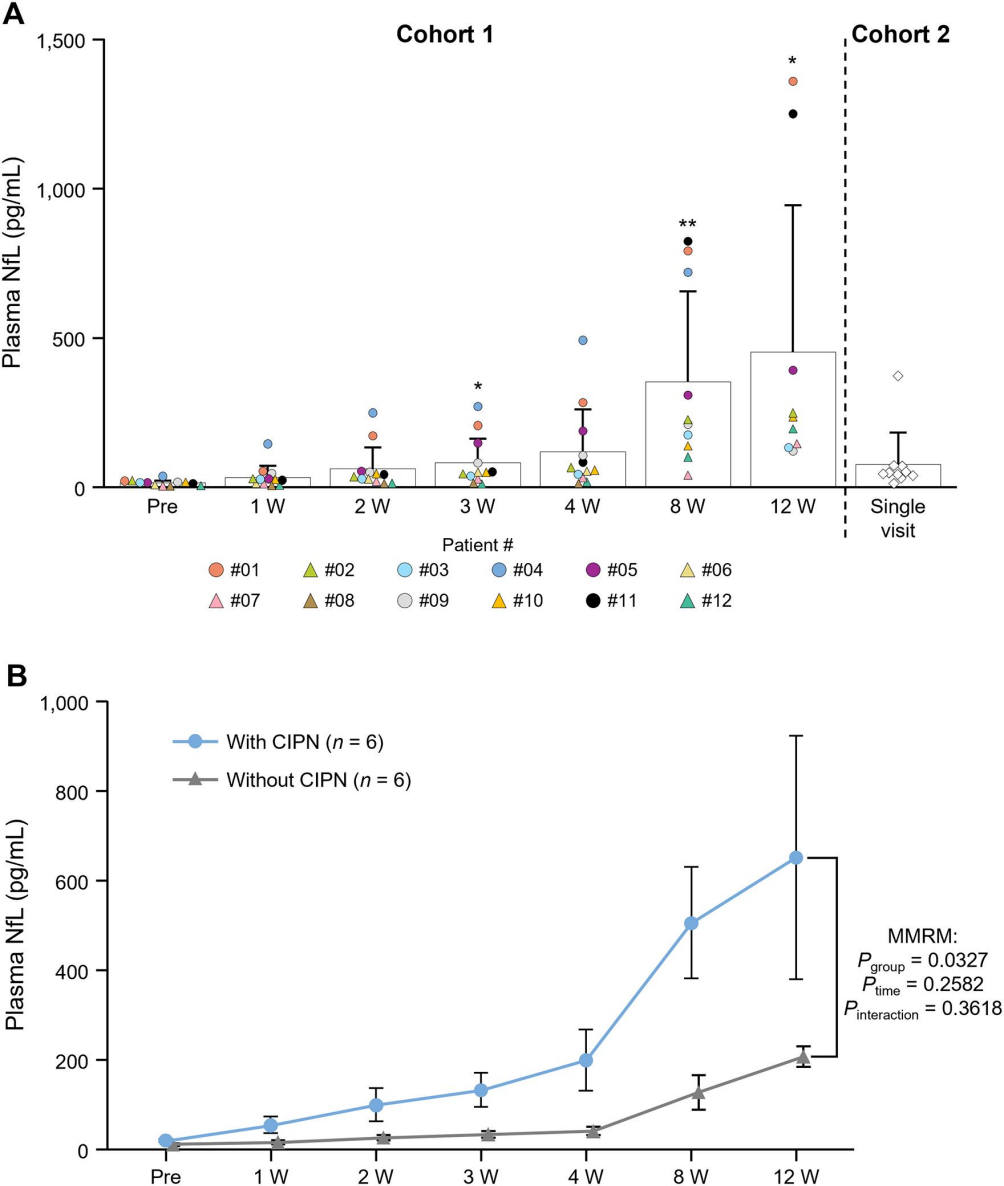
Plasma NfL levels from the start of paclitaxel chemotherapy (Cohort 1) and in patients with a prior diagnosis of CIPN (Cohort 2). **(A)** Changes in NfL levels over time in Cohort 1 and NfL levels measured at the single visit in Cohort 2. For Cohort 1, the colored symbols indicate the values for individual patients; circles represent patients with CIPN and triangles represent patients without CIPN. Bars represent the mean ± standard deviation. Comparisons between timepoints were made using the *t*-test with adjustment for multiple comparisons using the Holm method. *Adjusted *P* < 0.05 and **adjusted *P* < 0.01 vs. Pre. **(B)** Comparison of changes in NfL levels over time in patients with or without CIPN in Cohort 1. Values are the mean ± standard error. Linear MMRM was conducted with group, time, and an interaction of group × time as fixed factors, and subject as a random effect. When the group effect or the group × time interaction was significant, between-group comparisons at each timepoint were performed and the Holm method was used to adjust *P*-values for multiple comparisons across timepoints. Although the group effect was statistically significant, there were no statistically significant between-group differences at any timepoint. CIPN, chemotherapy-induced peripheral neuropathy; MMRM, mixed model for repeated measures; NfL, neurofilament light chain; Pre, pre-treatment; W, week.

Figure 2A illustrates the temporal changes in NfL levels following the initiation of paclitaxel treatment. A statistically significant increase in the NfL levels was observed beginning at approximately 3 weeks, which is earlier than the timepoint at which cases of CIPN occur, with a change of 68.00 pg/mL from the pre-treatment visit [95% confidence interval (CI), 18.60–117.40; *P* = 0.0092]. After applying the Holm method for multiple comparisons, the adjusted *P*-value was 0.0368. The NfL levels also showed significant increases at 8 and 12 weeks compared with the pre-treatment visit, with adjusted *P*-values of 0.0054 and 0.0280, respectively. By comparison, elevated NfL levels were not observed in Cohort 2. Figure 2B compares the NfL levels over time between six patients with CIPN and six patients without CIPN in Cohort 1. The increase in NfL levels was significantly greater in patients with CIPN than in patients without CIPN [MMRM: group, *P* = 0.0327; time, *P* = 0.2582; interaction (group × time), *P* = 0.3618]. Because the group effect was significant, between-group comparisons at each timepoint were performed using the MMRM, and adjusted for multiplicity across timepoints using the Holm method. However, no statistically significant between-group differences were observed at any timepoint with adjusted *P*-values of >0.05 at all timepoints.

### 3.4 Evaluation of galectin-3 as a candidate biomarker in Cohorts 1 and 2

**Figure 3:**
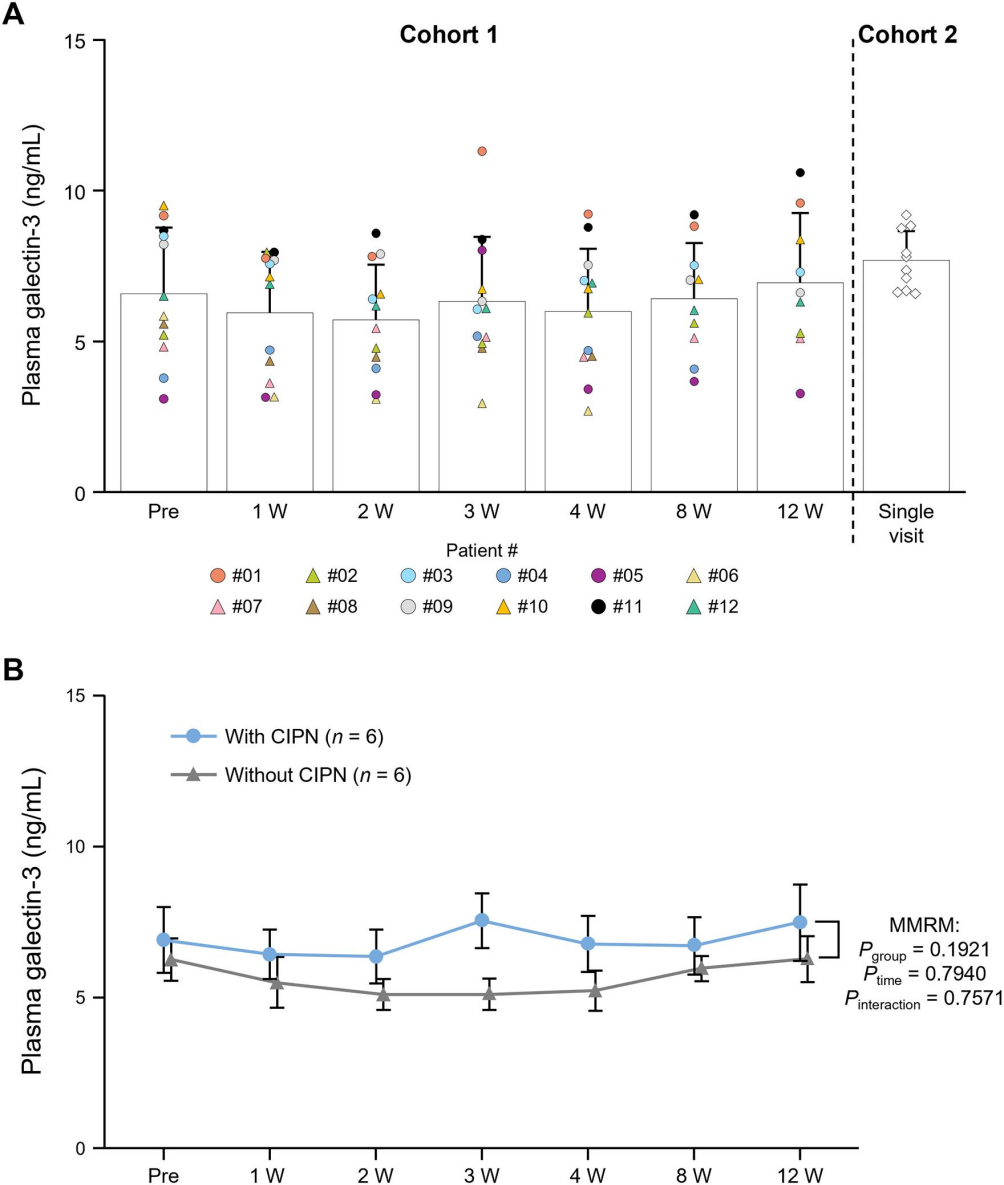
Plasma galectin-3 levels from the start of paclitaxel chemotherapy (Cohort 1) and in patients with a prior diagnosis of CIPN (Cohort 2). **(A)** Changes in galectin-3 levels over time in Cohort 1 and galectin-3 levels measured at the single visit in Cohort 2. For Cohort 1, the colored symbols indicate the values for individual patients; circles represent patients with CIPN and triangles represent patients without CIPN. Bars represent the mean ± standard deviation. Comparisons between timepoints were made using the *t*-test with adjustment for multiple comparisons using the Holm method. **(B)** Comparison of changes in galectin-3 levels over time between patients with or without CIPN in Cohort 1. Values are the mean ± standard error. Linear MMRM was conducted with group, time, and an interaction of group × time as fixed factors, and subject as a random effect. Because the group and group × time interaction were not statistically significant, between-group comparisons were not done. CIPN, chemotherapy-induced peripheral neuropathy; MMRM, mixed model for repeated measures; Pre, pre-treatment; W, week.

Figure 3A shows the temporal changes in plasma galectin-3 levels following the initiation of paclitaxel treatment. There were no significant increases in galectin-3 levels between the pre-treatment visit and 12 weeks of treatment in Cohort 1. Similarly, the galectin-3 levels in Cohort 2 were not significantly different to the pre-treatment levels in Cohort 1. Figure 3B illustrates the temporal changes in galectin-3 levels in six patients with CIPN and six patients without CIPN in Cohort 1. The changes in galectin-3 levels over time were not significantly different between the two groups in MMRM [group, *P* = 0.1921; time, *P* = 0.7940; interaction (group × time), *P* = 0.7571]. Because the group and group × time interaction were not statistically significant, between-group comparisons were not done.

### 3.5 Identification of proteins associated with CIPN using proteomic analysis

Table 2 shows the number of differentially expressed proteins in patients with and without CIPN that were identified by proteomic analysis. Proteins that commonly changed in both groups were not considered because they were likely to have changed due to paclitaxel. This allowed us to focus on candidate proteins that showed changes in their expression levels in the CIPN group using samples obtained at each timepoint. The numbers of proteins that were significantly upregulated with a fold-change >1.2 in all cases in the group and a *P*-value of <0.05 at 12 weeks relative to pre-treatment were 11 in patients with CIPN, 26 in patients without CIPN, and six were common to both groups. Therefore, five proteins were uniquely upregulated at 12 weeks in patients with CIPN. Conversely, eight proteins were significantly downregulated in patients with CIPN, six in patients without CIPN, and two were common to both groups, meaning six proteins were uniquely downregulated in patients with CIPN at 12 weeks relative to pre-treatment. Table 2 also lists the numbers of proteins that were upregulated or downregulated at 4 and 8 weeks relative to pre-treatment.

**Table 2:** Numbers of proteins that were upregulated or downregulated at 4, 8, or 12 weeks vs. pre-treatment in patients with or without CIPN in Cohort 1.

Number of differentially regulated proteins	Upregulated			Downregulated		
	At 4 W vs. Pre	At 8 W vs. Pre	At 12 W vs. Pre	At 4 W vs. Pre	At 8 W vs. Pre	At 12 W vs. Pre
Patients with CIPN	9	7	11	11	4	8
Patients without CIPN	19	15	26	10	9	6
Common to both groups	8	1	6	5	3	2
Only altered in patients with CIPN	1	6	5	6	1	6
Up/downregulated at all three timepoints	0			0		
Up/downregulated at two timepoints	1 (ADH4 at 8 and 12 W)			1 (VOPP1 at 4 and 12 W)		

Differentially regulated proteins were defined as those with a fold-change of ≥1.2 (upregulated) or ≤0.83 (downregulated) in all patients in the group, along with a *P*-value of <0.05 (paired *t*-test). Candidate selection was based on nominal *P*-values without controlling for the false discovery rate, meaning the number of identified proteins may include false positives.

ADH4, alcohol dehydrogenase 4; CIPN, chemotherapy-induced peripheral neuropathy; Pre, pre-treatment; VOPP1, vesicular overexpressed in cancer prosurvival protein 1; W, week.

Table 3 lists the proteins that were uniquely altered in patients with CIPN that were detected at each timepoint. No proteins were consistently upregulated across all three timepoints (weeks 4, 8, and 12). Among the upregulated proteins, alcohol dehydrogenase 4 (ADH4) was elevated at two timepoints. Although no proteins were consistently downregulated across all timepoints, vesicular overexpressed in cancer prosurvival protein 1 (VOPP1) was uniquely and consistently downregulated at two timepoints. The top three upregulated and downregulated proteins in patients with CIPN (regardless of timepoint) were: upregulated—ADH4, glutathione S-transferase alpha 2 (GSTA2), and alcohol dehydrogenase 1C (ADH1C); downregulated—Golgi SNAP receptor complex member 2 (GOSR2), cathelicidin antimicrobial peptide (CAMP), and neurexophilin-2 (NXPH2).

**Table 3:** Proteins that were uniquely upregulated or downregulated in patients with CIPN.

Timepoint	Protein		Fold-change	*P*-value (nominal)	BH *Q*-value	ST *Q*-value
Upregulated						
4 W	CXCL11	C-X-C motif chemokine ligand 11	1.36	0.00025	0.18564	0.08466
8 W	ADH1C	Alcohol dehydrogenase 1C	2.38	0.00418	0.60289	0.42288
	ADH4	Alcohol dehydrogenase 4	2.72	0.00281	0.60010	0.42092
	AKR1C4	Aldo-keto reductase family 1 member C4	2.05	0.01189	0.70037	0.49125
	GSTA2	Glutathione S-transferase alpha 2	2.45	0.00465	0.60289	0.42288
	H2BC21	Histone H2B type 1-O	1.98	0.02530	0.76303	0.53520
	MLN	Motilin	1.55	0.00131	0.56960	0.39953
12 W	ADH4	Alcohol dehydrogenase 4	1.41	0.00533	0.46268	0.40111
	C4A/C4B	Complement component 4A/4B	1.56	0.00436	0.46268	0.40111
	GBP1	Guanylate-binding protein 1	1.57	0.00237	0.46268	0.40111
	RPL5	Ribosomal protein L5	1.40	0.02995	0.57382	0.49745
	ZW10	Zeste white 10	1.49	0.00052	0.46268	0.40111
Downregulated						
4 W	CAMP	Cathelicidin antimicrobial peptide	0.64	0.00003	0.12046	0.05493
	CKB/CKM	Creatine kinase type B/M	0.69	0.01166	0.32692	0.14909
	PSD2	PH and SEC7 domain-containing protein 2	0.72	0.00016	0.17187	0.07838
	SCGB3A1	Secretoglobin family 3A member 1	0.68	0.00038	0.21065	0.09606
	TREM2	Triggering receptor expressed on myeloid cells 2	0.67	0.00362	0.29870	0.13622
	VOPP1	Vesicular overexpressed in cancer prosurvival protein 1	0.66	0.00097	0.21065	0.09606
8 W	NXPH2	Neurexophilin-2	0.64	0.00140	0.56960	0.39953
12 W	CDH11	Cadherin-11	0.72	0.00054	0.46268	0.40111
	COPE	Coatomer subunit epsilon	0.78	0.00427	0.46268	0.40111
	GOSR2	Golgi SNAP receptor complex member 2	0.62	0.01213	0.52816	0.45787
	NFKBIE	Nuclear factor kappa B inhibitor epsilon	0.68	0.00341	0.46268	0.40111
	NRN1L	Neuritin-like protein	0.80	0.000001	0.00445	0.00386
	VOPP1	Vesicular overexpressed in cancer prosurvival protein 1	0.69	0.00390	0.46268	0.40111

Proteins that were differentially regulated at two timepoints are indicated using underlined font. Proteins that were differentially regulated in both groups of patients are listed in Supplementary Table S2. *Q*-values were calculated simultaneously using the Benjamini–Hochberg and Storey methods, and provided for information purposes. Candidate selection was based on nominal *P*-values without controlling for the false discovery rate, meaning false positives may be included in this table.

BH, Benjamini–Hochberg; CIPN, chemotherapy-induced peripheral neuropathy; ST, Storey; W, week.

The proteins that were differentially regulated in the same directions in patients with and without CIPN are listed in Supplementary Table S2.

## 4 Discussion

In this study, we evaluated the exploratory role of NfL and galectin-3 as candidate biomarkers for CIPN in patients with breast cancer being treated with paclitaxel. We also sought to identify novel CIPN-associated proteins through proteomic analysis. Our findings revealed that NfL levels increased significantly in patients with CIPN after treatment initiation. Importantly, the NfL levels increased over time, beginning earlier than the onset of CIPN. Therefore, NfL may be a potential biomarker for early detection of CIPN. At individual timepoints, the between-group comparisons did not reach statistical significance after Holm adjustment. However, the overall group effect in MMRM was statistically significant, suggesting that NfL was consistently elevated in patients with CIPN throughout the observation period. A likely reason why the differences were not statistically significant at each timepoint is the limited sample size (*n* = 6 per group), which may have reduced the statistical power of the *post hoc* comparisons. Accordingly, these results should be interpreted as exploratory and need to be confirmed in larger prospective cohorts in the future. By contrast, galectin-3 levels demonstrated no significant temporal changes in either group of patients, and there were no discernible differences between patients with or without CIPN. The proteomic analysis identified several distinct proteins implicated in the pathogenesis of CIPN, including ADH4, which was upregulated, and VOPP1, which was downregulated in patients with CIPN, although these findings should be considered exploratory, as discussed later. We also observed signs of impaired nerve function (based on SNAP) in patients with CIPN.

These results provide novel insights into the molecular mechanisms underlying CIPN and underscore the clinical utility of NfL as a promising albeit candidate biomarker for early detection (13) and monitoring of the onset of CIPN due to the direct effects of paclitaxel on nerve axons and the release of NfL (14–16). Since the half-life of NfL was reported to range from approximately 20 to 43 days *in vivo* (16, 17), the discontinuation of paclitaxel will remove the injurious stimulus on the remaining nerve axons, and the NfL levels start declining towards the baseline levels within ∼3–6 months (14, 18). In Cohort 2, the NfL levels were close to the pre-treatment value in Cohort 1, which was likely due to the months-to-years interval following the completion of paclitaxel treatment. Because Cohort 2 represents a cross-sectional cohort of patients with established CIPN, these findings should be interpreted as supportive evidence of a biological association rather than validation of the predictive performance. Therefore, the NfL levels may reflect the extent of nerve damage during or shortly after treatment with paclitaxel in patients with CIPN. Importantly, our longitudinal human data combined with proteomic signatures provide an integrated view of how early axonal injury markers behave over time, highlighting the temporal separation between biochemical recovery (declining NfL) and persistent symptoms—an aspect that was insufficiently captured in prior studies. Our findings open new avenues for investigating the molecular pathways underlying CIPN and exploring potential therapeutic targets.

Previous studies on CIPN biomarkers predominantly relied on clinical evaluations and subjective patient-reported outcomes, highlighting a significant gap in the identification of objective molecular markers. Although prior studies have investigated NfL as a potential biomarker for CIPN in patients with breast cancer (9, 15), its clinical utility remains insufficiently explored. Paclitaxel is known to damage neurons through a variety of mechanisms, including axonal degeneration through microtubule stabilization, leading to impaired axonal transport and eventual neuronal damage (19), inhibition of axonal mRNA transport (20), as well as arrested distal growth (21). Elevated NfL levels may reflect the degree of axonal injury, providing a measurable indicator of the severity and progression of neuropathy. Our findings align with these earlier studies, providing additional evidence that elevated NfL levels are associated with neuronal damage in CIPN based on sensory nerve function, with our observation of a reduction in SNAP. Similarly, galectin-3 has been extensively studied in the context of inflammation and fibrosis (22, 23), and it is considered to be an important regulator of neuroaxonal damage in CNS-related diseases such as multiple sclerosis, Alzheimer's disease, and Parkinson's disease (24). However, its role in CIPN has not been established. Animal and cell-based studies point to a role of galectin-3 in regulating T-cell functions (22), and a pronociceptive effect by promoting macrophage infiltration in the pathogenesis of CIPN (10). Considering our findings, it remains to be elucidated whether galectin-3 is a useful biomarker for CIPN in humans. Mechanistically, paclitaxel-induced CIPN is considered a length-dependent axonopathy driven by microtubule stabilization and impaired axonal transport that preferentially affects distal peripheral axons rather than neuronal cell bodies or myelinating glial cells (25). In this context, NfL, a structural component of axons, is more likely to be directly released into the circulation following axonal injury, whereas galectin-3—primarily expressed in Schwann cells and immune-related cells—may reflect secondary glial or inflammatory responses that are not necessarily translated into measurable changes in circulating levels (26, 27). These differences in the cellular localization and release dynamics may help explain why changes in circulating NfL were more readily detectable than those of galectin-3 in our longitudinal human cohort, despite experimental evidence showing upregulation of galectin-3 in peripheral nerves following paclitaxel exposure (27).

Proteomic analysis has been sporadically used in CIPN research, and few studies have sought to identify CIPN-specific molecules or related pathways using proteomics-based methods (28, 29). Our study builds upon previous efforts by providing a comprehensive proteomic profile, in which we identified several proteins, such as ADH4 and VOPP1, that have not previously been linked to CIPN. In this analysis, considering the multiplicity of the omics analysis, we calculated *P-*values as well as *Q*-values using the Benjamini–Hochberg and Storey methods. However, because the number of samples used in the analysis was limited to 4–6 per timepoint, only a small number of proteins met the criteria based on fold-change and significance level of *P* < 0.05 at all timepoints. Therefore, we cannot rule out the possibility that some of the proteins identified in this study included false positives due to multiple testing.

ADH4, which was upregulated in patients with CIPN, is implicated in oxidative stress and cellular damage (30). Increased ADH4 levels may signify an adaptive response to paclitaxel-induced oxidative stress. Oxidative stress is a known contributor to neuronal damage (31, 32). Because ADH4 is involved in detoxification pathways, its upregulation could serve to protect cells (33). Notably, the proteomics analysis did not reveal any protein to be consistently up- or downregulated across all timepoints in patients with CIPN. This may reflect the dynamic and heterogeneous nature of CIPN, the limited sample size, or the inherent variability of plasma proteomics, including transient or low-abundance protein changes in relation to time-dependent biological processes. However, as an exploratory study, the findings may be useful for generating hypotheses in larger prospective studies.

We also observed consistent downregulation of VOPP1 in patients with CIPN, suggesting its involvement in pathways critical for neuronal survival. VOPP1 is involved in anti-apoptotic signaling and mitochondrial dysfunction (34). Its downregulation may compromise neuronal resilience to paclitaxel-induced stress. The role of VOPP1 in maintaining mitochondrial function is particularly relevant because mitochondrial dysfunction is a recognized mechanism in the pathogenesis of CIPN (35). Accordingly, the downregulation of VOPP1 could exacerbate neuronal vulnerability, suggesting it is a potential target for therapeutic interventions aimed at bolstering neuronal survival mechanisms. The differential expression of ADH4 and VOPP1 underscores the complex pathogenesis of CIPN that involves oxidative stress and impaired neuroprotective pathways. Taken together, these findings provide a nuanced understanding of the molecular processes that may contribute to CIPN, and suggest specific biological pathways—such as oxidative stress-related ADH4 upregulation and mitochondrial vulnerability associated with VOPP1 downregulation—that may serve as future therapeutic entry points, such as antioxidant modulation or mitochondrial-protective strategies. These observations are further supported by prior experimental studies demonstrating that modulation of oxidative stress and mitochondrial pathways can attenuate CIPN. For example, acetyl-L-carnitine was shown to prevent and reduce paclitaxel-induced peripheral neuropathy (19), and resveratrol was reported to exert antioxidant and anti-inflammatory effects in oxaliplatin-induced neuropathy models (36).

We assessed sensory nerve function during paclitaxel-based chemotherapy in Cohort 1 and in patients diagnosed with CIPN in Cohort 2. The apparent differences in SNAP between Cohorts 2 and 1 provide evidence of nerve dysfunction in patients diagnosed with CIPN in Cohort 2, in particular. Additionally, we observed a slight decrease in SNAP over time in Cohort 1, providing evidence of altered nerve function in these patients.

In the preceding section, we focused on ADH4 and VOPP1 because these proteins were found to be differentially regulated at two timepoints, suggesting those were persistent changes. Ten other proteins were found to be upregulated and 11 were downregulated at one timepoint. The clinical relevance of the differential expression of these proteins at a single timepoint is unclear, but there is evidence that they could be linked to CIPN. Owing to space constraints, exhaustive discussion of these proteins was not possible. However, we have briefly described the major biological roles and their relevance to CIPN or paclitaxel treatment in Supplementary Table S3. Further investigations of the biological relevance of these proteins may be of interest in the future.

Although our findings are promising, several limitations must be acknowledged. In particular, CIPN symptoms were assessed using CTCAE grading by healthcare professionals. This approach may have limited the sensitivity for detecting early or subclinical neuropathy, and may lead to underestimation of patient-reported symptoms. More sensitive assessment tools, such as patient-reported outcomes and quantitative sensory testing, are warranted. Additionally, patient-reported outcome measures could provide a more sensitive and comprehensive assessment of neuropathy (37, 38). In particular, despite performing robust analyses, the small sample size may limit the generalizability of our findings. The interval between treatment completion and study enrollment varied widely in Cohort 2, and may have influenced the biomarker levels and proteomic profiles. Larger, multicenter studies are needed to validate NfL and proteomic markers. The present study is also limited by the nature of the statistical analyses. We used paired *t*-tests to identify fold-changes in protein expression, without specifying a false discovery rate or applying multiplicity correction. Therefore, there is some risk of false positives, which may impact on the results. We also used MMRM to evaluate changes in NfL and galectin-3 levels over time, but the sample size and variability may mean the analyses were underpowered. Nevertheless, as an exploratory study, the results presented here may aid hypothesis generation in the future. Furthermore, although we identified novel proteins by proteomics analysis, it will be necessary to validate their roles in CIPN to establish causative links. We primarily sought to identify biomarkers and did not explore the mechanistic pathways. Future studies should integrate functional assays to elucidate the roles of the identified proteins.

In conclusion, this study supports NfL as a candidate biomarker for early detection and monitoring of neuropathy in patients with breast cancer being treated with paclitaxel. Additionally, the proteomic analysis identified several novel proteins, such as ADH4 and VOPP1, that warrant further investigation into their roles in the pathogenesis of CIPN. These findings provide deeper understanding of CIPN and a foundation for future biomarker-driven and therapeutic strategies. The identification of objective biomarkers, such as NfL, could significantly influence clinical practice, enabling timely interventions to mitigate CIPN and improve patient outcomes. Furthermore, our proteomics insights may inform the development of targeted therapies, ultimately advancing personalized medicine in oncology.

## Data Availability

The datasets generated and/or analyzed during this study are available from the corresponding author upon reasonable request by any qualified investigator. Proteomics data were deposited in the NCBI Gene Expression Omnibus under accession number GSE315782, through the following website: https://www.ncbi.nlm.nih.gov/geo/.
