# Supplementary material for: Exploratory identification of candidate biomarkers and molecular contributors to paclitaxel-induced peripheral neuropathy in patients with breast cancer through proteomic analysis
Source: Front Pain Res (Lausanne). 2026 Jun 16;7:1813700. doi: 10.3389/fpain.2026.1813700 (PMC13315020; doi:10.3389/fpain.2026.1813700)
Supplement: Supplementary file 1 [file Supplementaryfile1.docx]

# **Alt Text**

# **Figure 1 — Study Design**

Diagram of the study design for two patient cohorts, visually separating the two cohorts and listing the endpoints measured in each. Cohort 1 includes 12 patients with breast cancer who initiated weekly paclitaxel chemotherapy. Cohort 2 includes 10 patients with breast cancer who developed grade ≥2 peripheral sensory neuropathy during or after taxane-based chemotherapy. The study visits for Cohort 1 were at pre-treatment and at weeks 1, 2, 3, 4, 8, and 12 after starting taxane-based chemotherapy. Cohort 2 has a single evaluation visit. At each visit, the assessments included grading of peripheral sensory neuropathy using Common Terminology Criteria for Adverse Events version 5.0 Japan Clinical Oncology Group definition, sural sensory nerve conduction studies, measurement of plasma neurofilament light chain and galectin-3 levels, and plasma proteomics.

# **Figure 2a — Plasma NfL Over Time (Individual Patients)**

Bar chart with error bars and symbols depicting the plasma neurofilament light chain (NfL) levels in individual patients in Cohorts 1 and 2. The y-axis ranges from 0 to 1,500 pg/mL. Each bar and error bar represents the mean and standard deviation for 12 individual patients in Cohort 1 (measured at pre-treatment and at weeks 1, 2, 3, 4, 8, and 12) and in 10 patients in Cohort 2 (measured at a single timepoint). Each patient in Cohort 1 is represented by a symbol labeled #01 to #12. Several time points include asterisks indicating statistically significant differences compared with baseline or between cohorts. The graph shows the NfL levels tended to increase over time in Cohort 1, and the levels were generally higher in Cohort 1 than in Cohort 2.

# **Figure 2b — Plasma NfL by CIPN Status**

Line graph comparing plasma neurofilament light chain (NfL) levels between two subgroups of patients from Cohort 1, comprising 6 patients who developed chemotherapy-induced peripheral neuropathy (CIPN) and 6 patients who did not. The x-axis covers measurements taken pre-treatment and at weeks 1, 2, 3, 4, 8, and 12 after starting treatment. The y-axis ranges from 0 to 1,000 pg/mL. The lines indicate NfL levels increased over time in both groups, but the lines diverge and levels were consistently greater in patients with CIPN than in patients without CIPN. The figure visually suggests that NfL levels are generally greater in patients who developed CIPN during taxane therapy. The *P*-values determined using the mixed model for repeated measures (MMRM) are shown, with values of 0.0327 for the group effect, 0.2582 for the time effect, and 0.3618 for the interaction between group and time.

# **Figure 3a — Plasma Galectin-3 Over Time (Individual Patients)**

Bar chart with error bars and symbols depicting the plasma galectin-3 concentrations in individual patients in Cohorts 1 and 2. The y-axis ranges from 0 to 15 ng/mL. Each bar and error bar represents the mean and standard deviation for 12 individual patients in Cohort 1 (measured at pre-treatment and weeks 1, 2, 3, 4, 8, and 12) and in 10 patients in Cohort 2 (measured at a single timepoint). Each patient in Cohort 1 is represented by a symbol labeled #01 to #12. The graph displays small fluctuations in galectin-3 levels, with no clear or consistent changes over time in individual patients in Cohort 1, and no differences between Cohorts 1 and 2.

# **Figure 3b — Plasma Galectin-3 by CIPN Status**

Line graph comparing plasma galectin-3 levels between two subgroups of patients from Cohort 1, comprising 6 patients who developed chemotherapy-induced peripheral neuropathy (CIPN) and 6 patients who did not. The x-axis covers measurements taken pre-treatment and at weeks 1, 2, 3, 4, 8, and 12 after starting treatment. The y-axis ranges from 0 to 15 ng/mL. There were small changes in galectin-3 levels over time in both groups. The levels were slightly higher in patients with CIPN than in patients without CIPN at all timepoints. The figure visually shows that the galectin-3 levels do not differentiate CIPN status. The *P*-values determined using the mixed model for repeated measures (MMRM) are shown, with values of 0.1921 for the group effect, 0.7940 for the time effect, and 0.7571 for the interaction between group and time.

# **Supplementary Figure 1a — Sural Sensory Nerve Conduction Velocity**

Bar chart with error bars and symbols depicting the sural sensory nerve conduction velocity (SNCV), measured in meters per second (m/s), in individual patients in Cohorts 1 and 2. Each bar and error bar represents the mean and standard deviation for 12 individual patients in Cohort 1 (measured at pre-treatment and at weeks 1, 2, 3, 4, 8, and 12) and in 3 patients in Cohort 2 (measured at a single timepoint). Each patient in Cohort 1 is represented by a symbol labeled #01 to #12. The y-axis ranges from 0 to 70 m/s. The graph shows there were small fluctuations in SNCV with no clear or consistent changes over time in individual patients in Cohort 1, and no differences between Cohorts 1 and 2.

# **Supplementary Figure 1b — Sural Sensory Nerve Action Potential**

Bar chart with error bars and symbols depicting the sural sensory nerve action potential (SNAP), measured in microvolts, in individual patients in Cohorts 1 and 2. Each bar and error bar represents the mean and standard deviation for 12 individual patients in Cohort 1 (measured at pre-treatment and at weeks 1, 2, 3, 4, 8, and 12) and in 3 patients in Cohort 2 (measured at a single timepoint). Each patient in Cohort 1 is represented by a symbol labeled #01 to #12. The y-axis ranges from 0 to 35 microvolts. The graph shows the large variability in SNAP with no clear or consistent changes over time in individual patients in Cohort 1. SNAP tended to be lower at the single visit in Cohort 2 than at any timepoint in Cohort 1 although this did not reach statistical significance.
