# Supplementary material for: Exploratory identification of candidate biomarkers and molecular contributors to paclitaxel-induced peripheral neuropathy in patients with breast cancer through proteomic analysis
Source: Front Pain Res (Lausanne). 2026 Jun 16;7:1813700. doi: 10.3389/fpain.2026.1813700 (PMC13315020; doi:10.3389/fpain.2026.1813700)
Supplement: Supplementary file 2 [file Table1.docx]

Supplementary Material

**Exploratory identification of candidate biomarkers and molecular contributors to paclitaxel-induced peripheral neuropathy in patients with breast cancer through proteomic analysis**

Nobuyoshi Kittaka^1,2,*^, Hideaki Tahara^3,4^, Takashi Akazawa^3^, Yu Mizote^3^, Takashi Kitajima^5^, Keiichi Minami^6^, Yurika Sugano^2^, Takahiro Nakayama^2^

^1^Department of Breast Surgery, Osaka Rosai Hospital, 1179-3 Nagasone-cho, Kita-ku, Sakai-shi, Osaka 591-8025, Japan

^2^Department of Breast and Endocrine Surgery, Osaka International Cancer Institute, 3-1-69 Otemae, Chuo-ku, Osaka 541-8567, Japan

^3^Department of Cancer Drug Discovery and Development, Research Center, Osaka International Cancer Institute, 3-1-69 Otemae, Chuo-ku, Osaka 541-8567, Japan

^4^Center for Clinical Research, Osaka International Cancer Institute, 3-1-69 Otemae, Chuo-ku, Osaka 541-8567, Japan

^5^Translational Research, Ono Pharmaceutical Co., Ltd., 3-1-1 Sakurai, Shimamoto-cho, Mishima-gun, Osaka 618-8585, Japan

^6^Drug Discovery Technology, Ono Pharmaceutical Co., Ltd., 3-1-1 Sakurai, Shimamoto-cho, Mishima-gun, Osaka 618-8585, Japan

***Corresponding Author:** Nobuyoshi Kittaka
kittaka-no@osakah.johas.go.jp

| **Content** | **Page** |
| --- | --- |
| **SUPPLEMENTARY TABLE S1** Characteristics of Cohorts 1 and 2 | 2 |
| **SUPPLEMENTARY TABLE S2** List of proteins that were differentially regulated at any timepoint relative to pre-treatment in patients with and without CIPN | 3 |
| **SUPPLEMENTARY TABLE S3** Clinical/pathological relevance of the differentially regulated proteins identified in patients with CIPN | 3 |
| **SUPPLEMENTARY FIGURE S1** Results of nerve conduction tests from the start of paclitaxel chemotherapy (Cohort 1) and in patients with a prior diagnosis of CIPN (Cohort 2) | 8 |

**SUPPLEMENTARY TABLE S1** Characteristics of Cohorts 1 and 2

| **Characteristics** |  | **Cohort 1**  **(*N* = 12)** | **Cohort 2**  **(*N* = 10)** |
| --- | --- | --- | --- |
| Age (years) | Median (range) | 44 (30‒53) | 62 (50‒75) |
| BMI (kg/m²) | Median (range) | 21.1 (15.5‒28.2) | 20.9 (17.6‒28.1) |
| Menopausal status | Pre | 10 (83%) | 2 (20%) |
|  | Post | 2 (17%) | 8 (80%) |
| ECOG PS | 0 | 12 (100%) | 10 (100%) |
| Drinking history | Yes | 7 (58%) | 6 (60%) |
|  | No | 5 (42%) | 4 (40%) |
| Smoking history | Yes | 1 (8%) | 2 (20%) |
|  | No | 11 (92%) | 8 (80%) |
| Diabetes mellitus | No | 12 (100%) | 10 (100%) |
| Stage | I | 1 (8%) | 3 (30%) |
|  | IIA‒B | 6 (50%) | 5 (50%) |
|  | IIIA‒C | 5 (42%) | 2 (20%) |
| ER status | Positive | 4 (33%) | 4 (40%) |
|  | Negative | 8 (67%) | 6 (60%) |
| PgR status | Positive | 2 (17%) | 2 (20%) |
|  | Negative | 10 (83%) | 8 (80%) |
| HER2 status | Positive | 0 (0%) | 7 (70%) |
|  | Negative | 12 (100%) | 3 (30%) |
| Previous systemic therapy with taxane or platinum | No | 12 (100%) | 10 (100%) |

Values are *n* (%) of patients, unless otherwise stated.

BMI, body mass index; ECOG PS, Eastern Cooperative Oncology Group performance status; ER, estrogen receptor; HER2, human epidermal growth factor receptor 2; PgR, progesterone receptor; Post, post-treatment; Pre, pre-treatment.

**SUPPLEMENTARY TABLE S2** List of proteins that were differentially regulated at any timepoint relative to pre-treatment in patients with and without CIPN

| **Upregulated** |  |
| --- | --- |
| BCDIN3D | Pre-miRNA 5'-monophosphate methyltransferase |
| DCUN1D5 | DCN1-like protein 5 |
| FOXO1 | Forkhead box protein O1 |
| GGT2 | Inactive glutathione hydrolase 2 |
| HBQ1 | Hemoglobin subunit theta-1 |
| KRT1 | Keratin, type I cytoskeletal 1 |
| KRT5 | Keratin, type II cytoskeletal 5 |
| TP53I11 | Tumor protein p53-inducible protein 11 |
| IFIT2 | Interferon-induced protein with tetratricopeptide repeats 2 |
| TBCA | Tubulin-specific chaperone A |
| **Downregulated** |  |
| BPIFB1 | BPI fold-containing family B member 1 |
| CST6 | Cystatin-M |
| SCARA5 | Scavenger receptor class A member 5 |
| VNN2 | Pantetheine hydrolase VNN2 |

CIPN, chemotherapy-induced peripheral neuropathy.

**SUPPLEMENTARY TABLE S3** Clinical/pathological relevance of the differentially regulated proteins identified in patients with CIPN

| **Protein** | | **Clinical/pathological relevance** |
| --- | --- | --- |
| **Upregulated** | |  |
| ADH1C | Alcohol dehydrogenase 1C | Acetaldehyde generated by alcohol dehydrogenase (ADH) induces allodynia through transient receptor potential ankyrin 1 (TRPA1), and human Schwann cells express ADH/TRPA1/NADPH oxidase 1 (NOX1). Acute ethanol ingestion was shown to cause delayed mechanical allodynia in mice, and inhibition of ADH or deletion of TRPA1, a sensor for oxidative and carbonyl stress, prevented allodynia (1). |
| ADH4 | Alcohol dehydrogenase 4 | Acetaldehyde generated by ADH induces allodynia through TRPA1, and human Schwann cells express ADH/TRPA1/NOX1. Acute ethanol ingestion was reported to cause delayed mechanical allodynia in mice, and inhibition of ADH or deletion of TRPA1, a sensor for oxidative and carbonyl stress, prevented allodynia (1).  Single-nucleotide polymorphism (SNP) analysis of diabetes-related complications showed an association between SNP2 of the ADH4 gene and diabetic peripheral neuropathy (2). |
| AKR1C4 | Aldo-keto reductase family 1 member C4 | Unknown |
| C4A/C4B | Complement component 4A/4B | C4A was increased in cerebrospinal fluid and serum in patients with fibromyalgia (3). |
| CXCL11 | C-X-C motif chemokine ligand 11 | CXCL9, CXCL10, and CXCL11 expression was induced in Schwann cells, leading to the recruitment and infiltration of CD8+ T cells into tissues from patients with diabetic neuropathy (4). CXCL10 and CXCL11 expression was increased in the spinal cord in a murine model of neuropathic pain with potential roles in triggering neuropathy (5). |
| GBP1 | Guanylate-binding protein 1 | Unknown |
| GSTA2 | Glutathione S-transferase alpha 2 | Statins were reported to exhibit a class effect on ameliorating cytotoxic anti-cancer drug-induced mechanical allodynia by activating the GST pathway (6). GSTM1-null and GSTT1-null polymorphisms were associated with increased pain at 2 months after chemotherapy (7). |
| H2BC21 | Histone H2B type 1-O | Schwann cells deficient in Rnf40, which induces defective histone H2B monoubiquitination, arrested just before myelination or produced abnormally thin and unstable myelin, leading to peripheral neuropathy characterized by reduced myelination and progressive axonal degeneration (8). |
| MLN | Motilin | Acute pain was associated with increased expression of MLN and the MLN receptor in the spinal cord of a rat model of incisional pain (9). |
| RPL5 | Ribosomal protein L5 | Unknown |
| ZW10 | Zeste white 10 | Unknown |
| **Downregulated** | |  |
| CAMP | Cathelicidin antimicrobial peptide | In a rat model of postherpetic neuralgia, botulinum toxin ameliorated decreased CAMP expression and inhibited pain, and increased pyroptosis-associated protein and inflammatory factor expression in the rat dorsal root ganglion (10). |
| CDH11 | Cadherin-11 | N-cadherin, M-cadherin, and cadherin-11 were upregulated in a mouse sciatic nerve transection model. The transcripts were transiently upregulated in the distal stump of the neurotomized sciatic nerve during Wallerian degeneration. N-cadherin was abundant in myelinating Schwann cells during myelin degradation, whereas M-cadherin and cadherin-11 were potentially upregulated in proliferating Schwann cells (11). |
| CKB/CKM | Creatine kinase type B/M | Unknown |
| COPE | Coatomer subunit epsilon | Coatomer protein complex I (COPI) is a coatomer subunit involved in the translocation of δ-opioid receptor (DOPr), a receptor for ekaphalins involved in response to acute pain. By acting as binding partner to DOPr, COPI caused intracellular retention of DOPr in HEK293 cells (12). |
| GOSR2 | Golgi SNAP receptor complex member 2 | The Rab8a/SNARE pathway in astrocytes was reported to enhance vesicle trafficking and anchoring and increase secretion of bioactive molecules that may play an important role in the pathophysiology of neuropathic pain (13). |
| NFKBIE | Nuclear factor kappa B inhibitor epsilon | NFKBIE (IKKε) modulates neuropathic pain by activating nuclear factor-κB, and knockout of IKKε attenuated neuropathic pain in mice (14). Knockout of IKKε in mice strongly suppressed melanoma-associated pain, with concomitant decreases in the mRNA expression of pain-related genes, without affecting tumor growth compared to wild-type IKKε mice (15). |
| NRN1L | Neuritin-like protein | Neuritin is a neurotrophic factor that may mediate the antiapoptotic effect of hyperglycemia-induced insulin-like growth factor-1 on Schwann cells via PI3K and Bcl-2 (16). |
| NXPH2 | Neurexophilin-2 | Unknown |
| PSD2 | PH and SEC7 domain-containing protein 2 | Unknown |
| SCGB3A1 | Secretoglobin family 3A member 1 | Unknown |
| TREM2 | Triggering receptor expressed on myeloid cells 2 | TREM2 deficiency impaired energy metabolism and axon growth in the sciatic nerve, accompanied by exacerbation of neurologic deficits and suppression of nerve regeneration in a mouse model of acute motor axonal neuropathy (17). Administration of an anti-TREM2 neutralizing antibody significantly attenuated cisplatin-induced mechanical allodynia, sensory disturbance, and loss of intraepidermal nerve fibers (18). |
| VOPP1 | Vesicular overexpressed in cancer prosurvival protein 1 | Circulating Vopp1 and Vop1 mRNA were downregulated in the leukocytes of prenatal alcohol-exposed (PAE) rats with allodynia, but spinal cord circVop1 levels were upregulated in PAE rats with and without nerve injury (19). |

This table summarizes the main biological roles of each protein, and the proposed relevance to CIPN. These descriptions are not exhaustive and these proteins may be involved in other relevant pathways not mentioned here.

Unknown indicates no clear relevance could be identified by literature searches.

CIPN, chemotherapy-induced peripheral neuropathy.

**References**

1. De Logu F, Li Puma S, Landini L, Portelli F, Innocenti A, de Araujo DSM, et al. Schwann cells expressing nociceptive channel TRPA1 orchestrate ethanol-evoked neuropathic pain in mice. *J Clin Invest.* (2019) 129:5424‒41. doi: 10.1172/JCI128022

2. Mansour A, Mousa M, Abdelmannan D, Tay G, Hassoun A, Alsafar H. Microvascular and macrovascular complications of type 2 diabetes mellitus: exome wide association analyses. *Front Endocrinol (Lausanne).* (2023) 14:1143067. doi: 10.3389/fendo.2023.1143067

3. Gkouvi A, Tsiogkas SG, Bogdanos DP, Gika H, Goulis DG, Grammatikopoulou MG. Proteomics in patients with fibromyalgia syndrome: a systematic review of observational studies. *Curr Pain Headache Rep.* (2024) 28:565‒86. doi: 10.1007/s11916-024-01244-4

4. Tang W, Lv Q, Chen XF, Zou JJ, Liu ZM, Shi YQ. CD8(+) T cell-mediated cytotoxicity toward Schwann cells promotes diabetic peripheral neuropathy. *Cell Physiol Biochem.* (2013) 32:827‒37. doi: 10.1159/000354485

5. Piotrowska A, Rojewska E, Pawlik K, Kreiner G, Ciechanowska A, Makuch W, et al. Pharmacological blockade of CXCR3 by (±)-NBI-74330 reduces neuropathic pain and enhances opioid effectiveness - evidence from in vivo and in vitro studies. *Biochim Biophys Acta Mol Basis Dis.* (2018) 1864:3418‒37. doi: 10.1016/j.bbadis.2018.07.032

6. Aizawa F, Kajimoto H, Okabayashi A, Moriyama D, Yagi K, Takahashi S, et al. Statins ameliorate oxaliplatin- and paclitaxel-induced peripheral neuropathy via glutathione S-transferase. *Neurochem Int.* (2024) 180:105863. doi: 10.1016/j.neuint.2024.105863

7. Dunn PJ, Griffiths LR, Yates P, Haupt LM, Alexander KE. GSTM1 and GSTT1 polymorphisms associated with pain in a chemotherapy-induced peripheral neuropathy cohort. *J Cancer Res Clin Oncol.* (2023) 149:7405‒12. doi: 10.1007/s00432-023-04677-3

8. Wüst HM, Wegener A, Fröb F, Hartwig AC, Wegwitz F, Kari V, et al. Egr2-guided histone H2B monoubiquitination is required for peripheral nervous system myelination. *Nucleic Acids Res.* (2020) 48:8959‒76. doi: 10.1093/nar/gkaa606

9. Zhang Y, Zhao J, Hu N, Wang J, Chen X, Wang K, et al. Motilin and its receptor are expressed in the dorsal horn in a rat model of acute incisional pain: intrathecal motilin injection alleviates pain behaviors. *Front Neurosci.* (2023) 17:1104862. doi: 10.3389/fnins.2023.1104862

10. Wan Q. Botulinum toxin type A ameliorates rat dorsal root ganglia neuron pyroptosis in postherpetic neuralgia by upregulating cathelicidin antimicrobial peptide to inhibit neutrophil elastase. *Chem Biol Drug Des.* (2024) 103:e14406. doi: 10.1111/cbdd.14406

11. Padilla F, Marc Mège R, Sobel A, Nicolet M. Upregulation and redistribution of cadherins reveal specific glial and muscle cell phenotypes during Wallerian degeneration and muscle denervation in the mouse. *J Neurosci Res.* (1999) 58:270‒83. doi: 10.1002/(SICI)1097-4547(19991015)58:2<270::AID-JNR7>3.0.CO;2-Y

12. St-Louis É, Degrandmaison J, Grastilleur S, Génier S, Blais V, Lavoie C, et al. Involvement of the coatomer protein complex I in the intracellular traffic of the delta opioid receptor. *Mol Cell Neurosci.* (2017) 79:53‒63. doi: 10.1016/j.mcn.2016.12.005

13. Xiao Y, Wang G, He G, Qin W, Shi Y. Rab8a/SNARE complex activation promotes vesicle anchoring and transport in spinal astrocytes to drive neuropathic pain. *Biomol Biomed.* (2024) 24:1290‒1300. doi: 10.17305/bb.2025.12773. Erratum in: *Biomol Biomed*. (2025) 25:2585. doi: 10.17305/bb.2025.12773

14. Möser CV, Möller M, Fleck SC, Thomas D, Geisslinger G, Niederberger E. Inhibition of the protein kinase IKKepsilon attenuates neuropathic pain in mice. *Neuropharmacology.* (2019) 146:198‒211. doi: 10.1016/j.neuropharm.2018.12.004

15. Möser CV, Meissner M, Laarmann K, Olbrich K, King-Himmelreich TS, Wolters MC, et al. The protein kinase IKKepsilon contributes to tumour growth and tumour pain in a melanoma model. *Biochem Pharmacol.* (2016) 103:64‒73. doi: 10.1016/j.bcp.2015.12.016

16. Yan L, Xie M, Lu H, Zhang H, Shi M, Zhang Y, et al. Anti-apoptotic effect of IGF1 on Schwann exposed to hyperglycemia is mediated by neuritin, a novel neurotrophic factor. *Mol Neurobiol.* (2018) 55:495‒505. doi: 10.1007/s12035-016-0331-3

17. Zhang N, Ji Q, Chen Y, Wen X, Shan F. TREM2 deficiency impairs the energy metabolism of Schwann cells and exacerbates peripheral neurological deficits. *Cell Death Dis.* (2024) 15:193. doi: 10.1038/s41419-024-06579-9

18. Hu LY, Zhou Y, Cui WQ, Hu XM, Du LX, Mi WL, et al. Triggering receptor expressed on myeloid cells 2 (TREM2) dependent microglial activation promotes cisplatin-induced peripheral neuropathy in mice. *Brain Behav Immun.* (2018) 68:132‒45. doi: 10.1016/j.bbi.2017.10.011

19. Noor S, Pritha AN, Pasmay AA, Sanchez JE, Sanchez JJ, Fernandez-Oropeza AK, et al. Prenatal alcohol exposure dysregulates spinal and circulating immune cell circular RNA expression in adult female rats with chronic sciatic neuropathy. *Front Neurosci.* (2023) 17:1180308. doi: 10.3389/fnins.2023.1180308

**
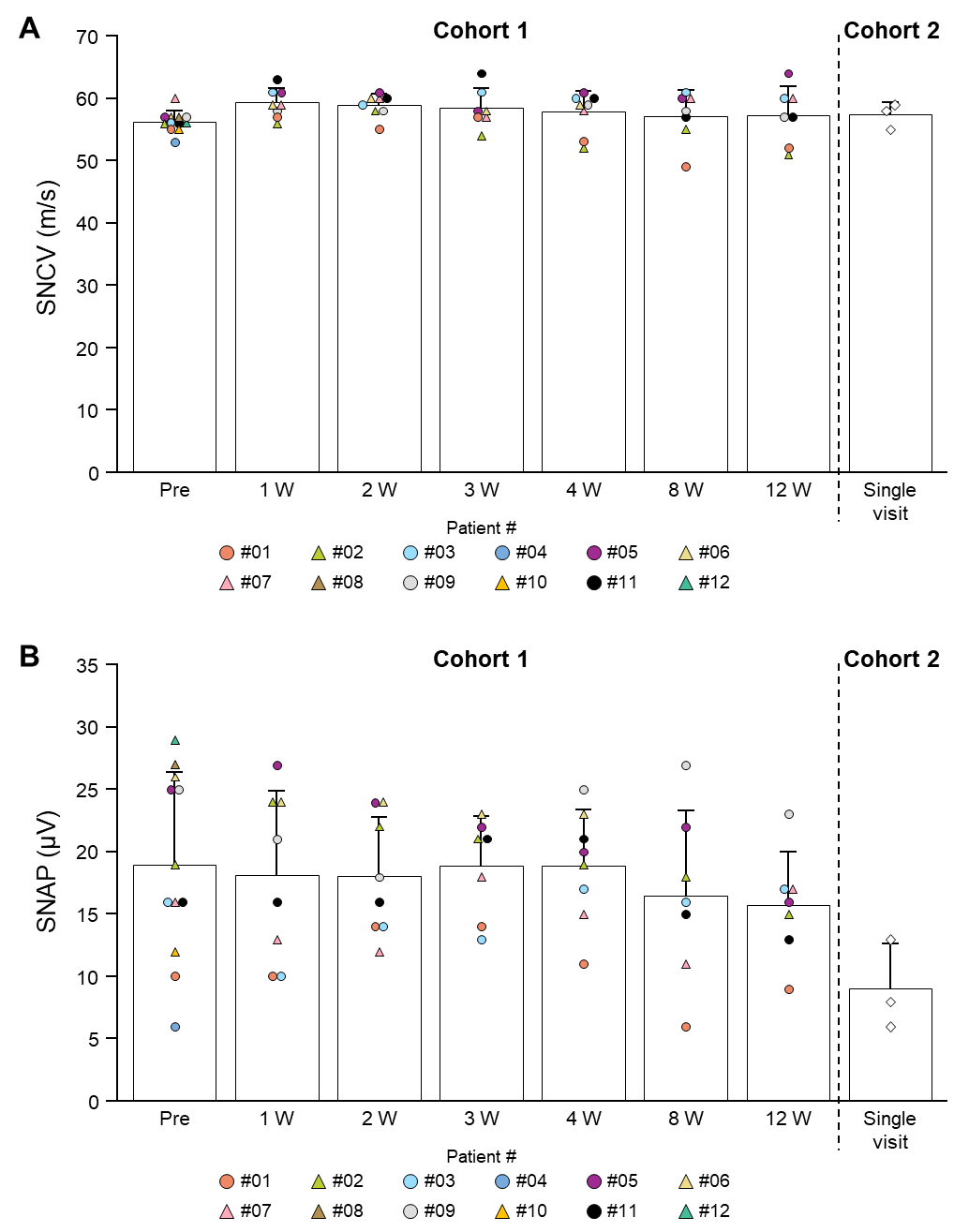
**

**SUPPLEMENTARY FIGURE S1** Results of nerve conduction tests from the start of paclitaxel chemotherapy (Cohort 1) and in patients with a prior diagnosis of CIPN (Cohort 2). (A,B) Changes in SNCV (A) and SNAP (B) of the sural nerve over time in Cohort 1 and recorded at the single visit in Cohort 2. For Cohort 1, the colored symbols indicate the values for individual patients; circles represent patients with CIPN and triangles represent patients without CIPN. Bars represent the mean ± standard deviation. CIPN, chemotherapy-induced peripheral neuropathy; Pre, pre-treatment; SNAP, sensory nerve action potential; SNCV, sensory nerve conduction velocity; W, week.
